# A visual method for direct selection of high-producing *Pichia pastoris *clones

**DOI:** 10.1186/1472-6750-11-23

**Published:** 2011-03-21

**Authors:** Fan Hu, Xin Li, Jie Lü, Pei Hong Mao, Xiang Jin, Ben Rao, Peng Zheng, Yu Lin Zhou, Sheng Yi Liu, Tao Ke, Xiang Dong Ma, Li Xin Ma

**Affiliations:** 1 Hubei Key Laboratory of Industrial Biotechnology, College of Life Science, Hubei University, Wuhan, 430062, PR China; 2 Oil Crops Research Institute, Chinese Academy of Agricultural Sciences, Wuhan, 430062, PR China; 3 Institute of Ion Beam Biotechnology, College of Physics Science and Technology, Xinjiang University, Urumqi, 830008, PR China; 4 College of Life Science and Technology, Nanyang Normal University, Nanyang 473061, PR China

## Abstract

**Background:**

The methylotrophic yeast, *Pichia pastoris*, offers the possibility to generate a high amount of recombinant proteins in a fast and easy way to use expression system. Being a single-celled microorganism, *P. pastoris *is easy to manipulate and grows rapidly on inexpensive media at high cell densities. A simple and direct method for the selection of high-producing clones can dramatically enhance the whole production process along with significant decrease in production costs.

**Results:**

A visual method for rapid selection of high-producing clones based on mannanase reporter system was developed. The study explained that it was possible to use mannanase activity as a measure of the expression level of the protein of interest. High-producing target protein clones were directly selected based on the size of hydrolysis holes in the selected plate. As an example, the target gene (9elp-hal18) was expressed and purified in *Pichia pastoris *using this technology.

**Conclusions:**

A novel methodology is proposed for obtaining the high-producing clones of proteins of interest, based on the mannanase reporter system. This system may be adapted to other microorganisms, such as *Saccharomyces cerevisiae *for the selection of clones.

## Background

Recently, the methylotrophic yeast *Pichia pastoris *is used for high level production of recombinant proteins for basic research and medical applications [1-4]. Many heterologous proteins have been successfully expressed in *Pichia pastoris *due to its exceptional natural capacity for heterologous protein production http://www.kgi.edu/Faculty-and-Research/James-M-Cregg.html. *Escherichia coli *can produce large amounts of heterologous protein, but due to the difference in protein folding environment and inability to perform posttranslational modifications, it fails to deliver correctly folded functional protein. Whereas *Pichia pastoris *eukaryotic system has been used successfully for non-expressible proteins in *E. coli *[5]. The main advantage of *Pichia *over *E. coli *is to produce disulfide bonds and glycosylations in proteins; this means that in cases where disulfides are necessary, *E. coli *might produce a misfolded protein that is usually inactive or insoluble.

Yeasts differ substantially from higher eukaryotes in post-translational modification, especially in glycosylation. *Saccharomyces cerevisiae *glycosylation pattern usually results in hyperglycosylated inactive proteins [6,7]. *Pichia pastoris *strains have been re-engineered to overcome this undesired glycan modification [5]. Availability of new strains with mammalian-type glycosylation capabities has made *P. pastoris *more important for the industrial production of therapeutic proteins [8]. Optimization of protein yield, which is primarily determined by protein expression levels, is a very important cost-saving area. A good screening and selection method for high-producing *Pichia pastoris *clones certainly help to lower the costs significantly.

The major method to select the high-producing recombinants in yeast is to use the antibiotic resistance genes. Mostly yeast expression vectors contain dominant drug resistance markers which allow enrichment of strains with multiple copies of foreign gene expression cassettes. It is deliberated that vectors contains the bacterial *kanamycin *resistance gene confer dose-dependent resistance to the antibiotic G418 in *P. pastoris *transformants [9], whereas some series of vectors which contains the *Sh ble *gene confer dose-dependent resistance to the antibiotic Zeocin. Selection of multi-copy transformants is usually performed, after the primary selection of transformants on plates, containing different concentrations of antibiotic. However, there are limitations of this method as: (*i*) the number of colonies screened by antibiotic is limited to approximately 100, *(ii) *The higher vector copy number does not always result in the higher protein yield, the protein expression level still needs to be determined. So, additional methods such as western blot analysis [9] or enzyme-linked immunosorbent assay (ELISA) [10,11] are in dire need to determine the expression level of the protein. Consequently, more versatile system are required for rapid selection of high-producing clones.

In this critique, a novel method for rapid selection of high-producing clones based on the mannanase activity reporter system is emphasized. The role of target protein 9Elp-Hal18 has not been obviously studied in the *P. pastoris*. During the study a gene encoding 'mannanase' was selected as the reporter gene that was to be expressed with the gene for target protein 9Elp-Hal18, linked by short peptides Glu-Lys-Arg-Glu-Ala-Glu-Ala (EKREAEA) (recognized by the Kex2 and Ste13 protease) was investigated.

## Results

### Construction of the recombinant expression vector pHBM306

**Figure 1:**
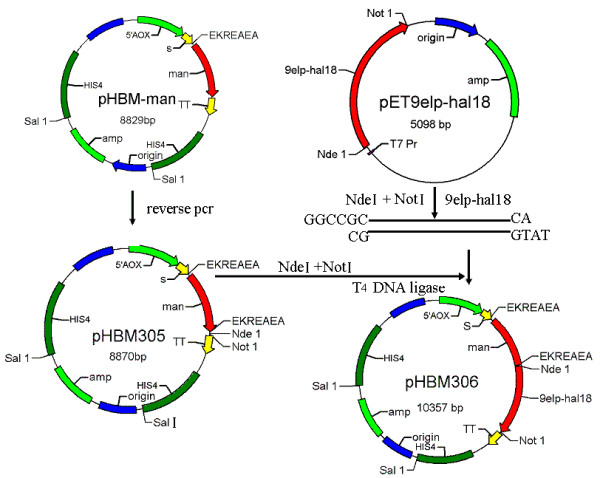
**Flow chart of construction of the recombinant *Pichia pastoris *vector pHBM306**.

The recombinant plasmid pHBM305 was constructed by reverse PCR. The sequence of short peptides Glu-Lys-Arg-Glu-Ala-Glu-Ala (EKREAEA) and the restriction enzyme sites was confirmed by Invitrogen Company. pHBM306 was constructed by inserting 9elp-hal18 gene between the *Nde *I and *Not *I restriction sites. Flow chart is given in Figure 1.

### Selection of the clones of different sizes for the mannanase activity analysis

**Figure 2:**
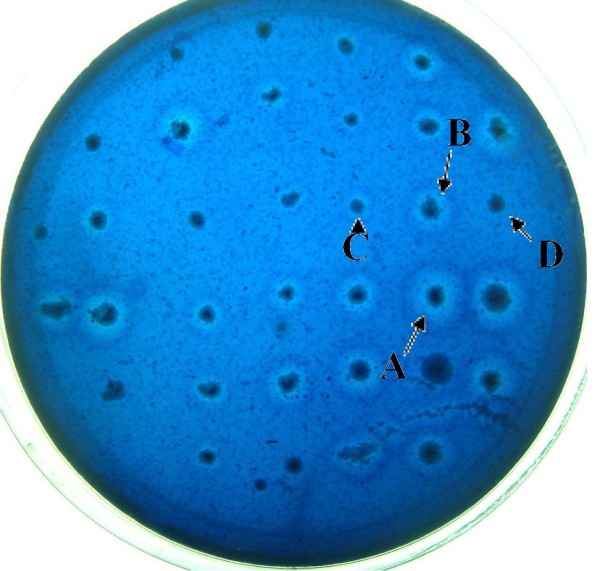
**Different size of hole after 24 hours culture in the BMMY plate**.

**Figure 3:**
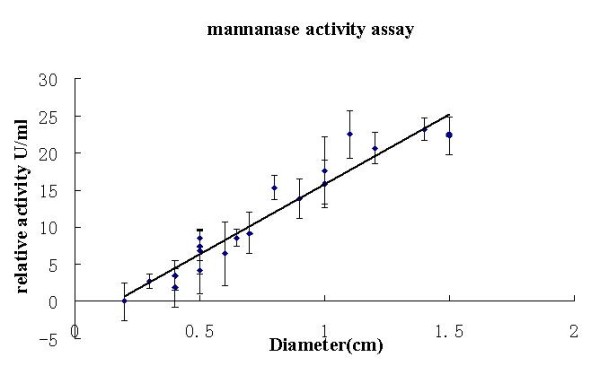
**Relative activity assay of the fermentation broth supernatant**. All measurements were carried out as triplicates, and means ± SD are shown. The trend line shows a linear; Curve fitting was performed by Microsoft Excel 2003.

*P. pastoris *GS115 was transformed with linearized pHBM306 plasmid DNA and the recombinant transformants were first selected on minimal selective MD medium. These clones were transferred to the BMMY plates containing thypan blue which makes the plates look blue colored. Clear zone can be seen around mannanase expressing colonies which could hydrolyze the Konjac Glucomannan (KGM). Different sizes of hydrolysis holes were observed and measured after 24-48 hours of culturing (Figure 2). Twenty clones with different sizes of hydrolysis holes were measured and selected for the following enzyme activity assay. It is observed from the mannanase activity analysis that clones with larger clear zone correlate with higher mannanase activity (Figure 3).

### Purification, characterization and SDS-PAGE analysis of fusion protein

**Figure 4:**
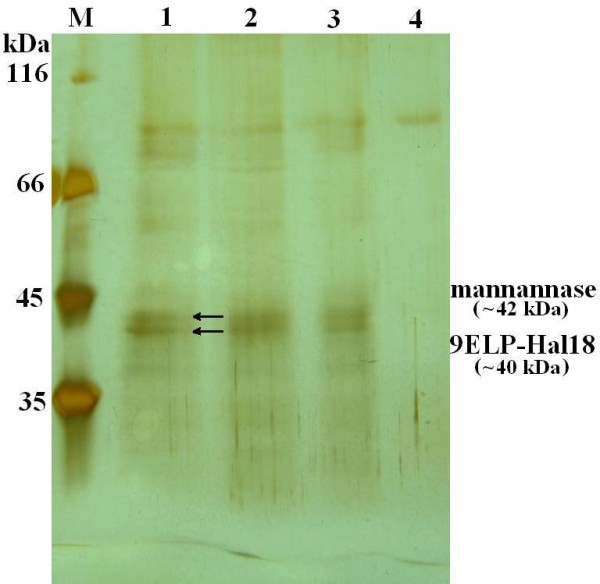
**SDS-PAGE analysis of supernatant of fermentation broth at each stage**. M, molecular weight markers; 1, supernatant of clone A; 2, supernatant of clone B; 3, supernatant of clone C; 4, supernatant of clone D (control).

**Figure 5:**
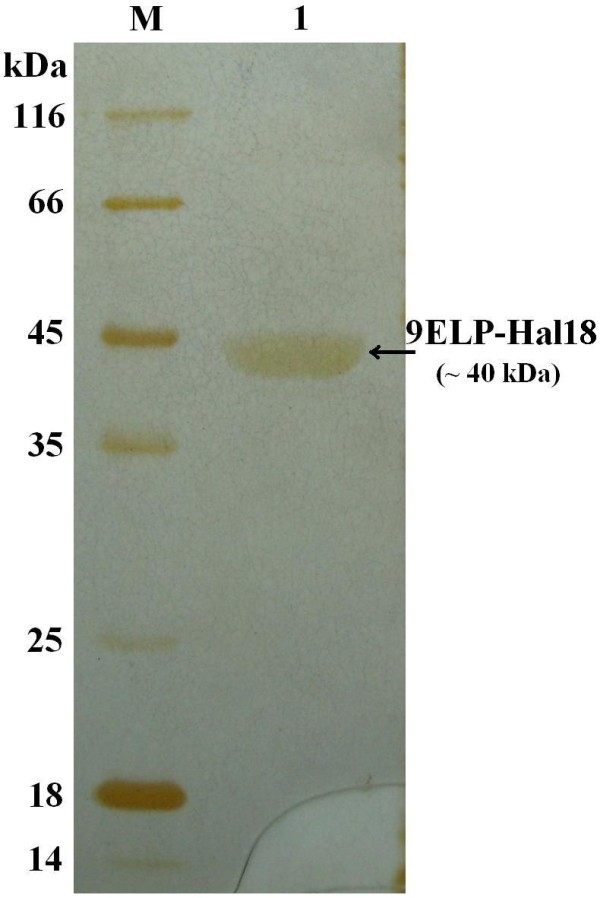
**SDS-PAGE analysis of purified 9Elp-Hal18**. M, molecular weight markers; 1, purified the protein 9Elp-Hal18.

For further confirmation, four clones with different diameter of holes for the following mannanase activity assay and target protein assay were selected. The supernatant of each fermentation broth was analyzed by SDS-PAGE after 120 hours of culturing. Results clearly demonstrate successful separation of the reporter and target protein with molecular mass evaluation of ~42 kDa and ~40 kDa, respectively (Figure 4) and are well in consistence with the actual molecular masses. The target protein was also purified from the supernatant of fermentation broth by inverse transition cycle (ITC) procedure. The SDS-PAGE assay results demonstrated 9Elp-Hal18 was successfully purified with good purity (Figure 5).

Mannanase activity measurement was also executed for the clones selected in order to determine the quality of the target protein 9Elp-Hal18 which was purified from the supernatant of cultures. About 448.5 mg/L of 9Elp-Hal18 with 30 U/ml of mannanase activity(A), 336.7 mg/L of 9Elp-Hal18 with 26 U/ml of mannanase activity, 275.3 mg/L of 9Elp-Hal18 with 20 U/ml of mannanase activity (C) and 0.23 mg/L of 9Elp-Hal18 with 1.23 U/ml of mannanase activity (D), respectively were purified. These results clearly support our hypothesis that larger clear zone corresponds to high production of the target protein.

## Discussion

The methylotrophic yeast *P. pastoris *has already been used successfully for the production of various recombinant heterologous proteins [12,13] and has been licensed to more than 120 companies in the biotechnological, pharmaceutical, animal health and food industries http://www.pichia.com. Some of the proteins are produced at grams per liter levels. Using the *P. pastoris *system to express foreign proteins has many advantages: The techniques needed for molecular genetic manipulation are similar to those well established for *S. cerevisiae*; *P. pastoris *can be easily grown to high cell densities using defined minimal media and is able to introduce eukaryotic posttranslational modifications; Distribution and chain length of N-linked oligosaccharides are different from those of *S. cerevisiae*. For instance, the chain length is significantly shorter, making it an interesting alternative for the extra-cellular expression of human proteins [14]; Complex proteins such as human glycoproteins or human collagens are also successfully processed and secreted by *P. pastoris *[15-18]; Another advantage of the *P. pastoris *expression system is the secretion of foreign proteins into culture media which making it convenient to obtain large amounts of foreign proteins in a relatively pure form without the contamination of cellular proteins. With the availability of new genetically engineered strains that show mammalian-type glycosylation, interest in using *P. pastoris *for the industrial production of therapeutic proteins has increased even further. So a good method for the screening and selection of high-producing transformants is certainly very useful that will ultimately decrease the cost of the industrial production.

The concept of a mannanase reporter system is to use the mannanase activity as a measure of the expression levels of a protein of interest. This can be achieved by integrating the reporter gene (mannanase) and the gene of interest under the same promoter, from where they can be expressed proportionally. We cloned the fusion protein expression cassette into the pHBM305 vector, resulting in pHBM306 expression vector (Figure 1). In both the pHBM305 and the modified pHBM306 plasmids, the HIS4 selection marker was used for selection of stable integrants based on complementation of *P. pastoris *HIS4 mutant strains. For the fusion protein expression, the AOX1 promoter was used for expression of the protein of interest [19]. During the protein secretion pathway, the fusion protein was separated by the cleavage of the kex2 and ste13 protease. Results also show that the marker protein is separated from the target protein (Figure 4) and the mannanase activity is proportional to the target protein expression level. The enzyme activity assay also clearly showed that the bigger hydrolysis hole is correlated with high activity of clone. It is concluded that the activity of the mannanase or the size of hydrolysis hole is a measure of the 9Elp-Hal18 expression because it has a positive correlation with quantity of the target protein.

Compared with other selection methods, such as using antibiotics, G418 or Zeocin for selecting the multi-copy clones, the mannanase reporter system has several advantages in screening high-producing clones. First, it's bio-safe and eliminates the need for antibiotics as well as of enzyme-linked immunosorbent assay and western blot analysis, which are time-consuming and expensive techniques. Second, it is a high-throughput selection method, which can efficiently select thousands of clones at one time, in contrast with the antibiotic screening, which is limited to approximately 100 clones at a time. Third, it can be screened and selected directly by the clear zones or hydrolysis holes in the plate. The bigger holes corresponding to high-producing clones. Also this screening system has potential to give more output [20] when combined with multi-copy in vivo fusion of the genes in a head to tail pattern. We intend to conduct these experiments in our future research work.

When deciding that protein secretion is the preferred approach to be used for a particular target protein, a choice of the specific secretion signal must be made [20]. This selection can be based on the protein's own native secretion signal (if it has one), the *S. cerevisiae *alpha -mating factor pre -pro leader sequence (α-MF), the acid phosphatase signal sequence (PHO) or the invertase signal sequence (SUC2) [20,21]. The most commonly used signal sequence in *P. pastoris *secretion systems is the *S. cerevisiae *alpha -mating factor pre -pro leader sequence. Although the α-MF pre -pro sequence has been used extensively. There are some instances where its application has been problematic. The main reason is due to the insertion of Glu-Ala dipeptide repeats between the amino-terminus of the mature recombinant protein and the Kex2p cleavage site. Although the Glu-Ala repeats do improve the proportion of correctly cleaved material by preventing steric hindrance of the kex2p cleavage site [20,22]. Their presence results in the generation of a recombinant protein with Glu-Ala amino terminal extensions. This can lead to N-terminal ragged ends due to differential amino-terminal processing because of the inability of the ste13p to process the large quantity of recombinant protein that is being produced [20,23]. As the presence of these amino-terminal dipeptide extensions is undesirable for many recombinant protein applications, the Glu-Ala repeats between the mature protein and the kex2p can be omitted as part of the cloning approach in order to achieve a more authentic amino-terminus. Omission of these Glu-Ala repeats, however, can result in a decreased efficiency in the Kex2p cleavage specificity. In this case, secretion of the pro-protein cleavage products that have long amino-terminal extensions corresponding to 9-11 amino acids of the α-MF pro-region has been observed [20,24]. This can be an issue when producing biopharmaceuticals. The one possible way to solve this problem is to treat fermentation broth by adding the purified Kex2 protease in vitro [25].

## Conclusions

In heterologous protein production, fast and inexpensive screening processes are desperately required to reduce the production cost of recombinant proteins. The *Pichia pastoris *products will eventually appear in the market and the use of this mannanase reporter system is obviously a valuable substitution method for time consuming and expensive selection techniques. The results clearly demonstrate that this visual method brings about rapid selection of high-producing clones for expression of secretary recombinant proteins. Furthermore, such reporter system can be easily applied to other industrially important yeast expression systems.

## Methods

### Strains and plasmids

*E. coli *XL10-GOLD (Stratagene, La Jolla, CA) was used to construct the recombinant plasmids. *P. pastoris *GS115 strain, genotype *his4 *(Invitrogen, Carlsbad, CA, USA), was used for yeast transformation and protein expression. pHBM-man [obtained from our own lab] was used to construct the expression vector pHB306. Vector pET-9elp-hal18 constructed in a previous research work contained the gene for the target protein (9Elp-Hal18) and Konjac Glucomannan (KGM) was used as the mannanase hydrolysis substrate.

### Construction of expression plasmid

Short peptides Glu-Lys-Arg-Glu-Ala-Glu-Ala and two restriction sites, *Nde *I and *Not *I were added at the ends of the mannanase gene by reverse PCR. Product of 9elp-hal18 gene was selected as the target protein to evaluate the new reporter system. 9elp-hal18 gene was obtained from the plasmid pET-9elp-hal18 by restriction digestion at the *Nde *I and *Not *I sites. These were cloned between the same restriction sites in the plasmid pHBM305. This new recombinant plasmid was designated as pHBM306.

### Yeast Transformation

pHBM306 plasmid DNA (10 μg) was linearized by using *Sal *I restriction enzyme to delete ampicillin resistance gene and the origin of *E. coli*. *P. pastoris *cells were transformed with the linearized plasmid DNA by electroporation method according to the protocol given in *Pichia *Expression kit manual. Yeast cells were grown in yeast extract-peptone-glucose (YPD) medium following the manufacturer's recommendations. Plasmid pHBM905 was used to prepare the control strain.

### Mannanase Activity Assay of the clones with different sizes of clear zone

Linearized pHBM306 was transformed into *P. pastoris *GS115 and integrated at the *P. pastoris *HIS4 locus. Transformants were first selected on minimal selective MD medium (0.34% YNB, 4×10^-5 ^% biotin, 1% dextrose, and 1.5% agar) and then transferred to the BMMY plates (100 mM potassium phosphate, pH 6.0, 0.34% YNB, 4×10^-5 ^% biotin, 0.5% methanol, 1% yeast extract, 2% peptone) containing 1% KGM and 0.02% Thypan blue. After 24-48 hours, hydrolysis holes of different sizes could be observed in the culture plates. Twenty clones with different sizes of hydrolysis holes were measured and selected for the following enzyme activity assay.

Prior to mannanase activity measurement on plate directly, twenty clones with different sizes were selected from BMMY with KGM added and were inoculated in 50 ml BMGY medium (1% glycerol replaced by 0.5% methanol in BMMY) in a 500 ml shake flask. These cultures were centrifuged when optical density reached 20 (equivalent to 1×10^9 ^cells/ml), at 600 nm (OD_600_). The cell mass was re-suspended to 25 ml BMMY medium to induce recombinant mannanase expression. These cultures were grown for 120 hours at a temperature of 28°C while shaking at 250 rpm. Mannanase activity in culture supernatants was assayed after 120 hours culture. It was determined by measuring the amount of reducing sugars liberated during the hydrolysis of KGM by the dinitrosalicylic acid method [26]. The standard assay reaction mixture consisted of 3% (w/v) of polysaccharide substrates supplemented with 50 mM sodium citrate buffer (pH 6.0) and enzyme to make a final volume of 0.3 ml. The reaction mixture was incubated at 55°C for 15 min because one unit of enzyme produced 1 μmol of reducing sugar per min.

### Purification, characterization and SDS-PAGE analysis of 9Elp-Hal18

Target protein concentration was also measured for the colonies selected to determine Mannanase activity. Target protein 9Elp-Hal18 was purified from the supernatant of the cultures after 120 hours. Due to the unique properties of Elp fusion protein, 9Elp-Hal18 can be easily separated and purified from mannanase and other contaminating proteins, from the supernatant by inverse transition cycle (ITC) procedure (a very simple method for purification of recombinant proteins) [27,28]. Target protein was purified from the supernatant of fermentation broth was mixed with protein sample buffer (0.5 M Tris-HCl [pH 6.8], 10% glycerol, 5% sodium dodecyl sulfate [SDS], 5% β-mercaptoethanol, 0.25% bromophenol blue), heated to 100°C for 5 min and then subjected to 12% (w/v) SDS-polyacrylamide gel electrophoresis (SDS-PAGE). The protein bands were detected by silver staining. Protein concentrations were measured using Micro-BCA Protein Assay Reagent (Pierce, USA).

## Authors' contributions

FH, XL, conceived of the study, participated in its design and carried out the molecular genetic studies; JL, participated in the sequence alignment and draws the vector maps; PHM, XJ performed the statistical analysis; BR, PZ carried out the enzyme activity assay; YLZ, SYL, TK, have been involved in drafting the manuscript and revising it critically for important intellectual content; XDM, LXM have given final approval of the version to be published. All authors read and approved the final manuscript.
